# Crystal structure of spinel-type Li_0.64_Fe_2.15_Ge_0.21_O_4_

**DOI:** 10.1107/S205698901600414X

**Published:** 2016-03-15

**Authors:** Günther J. Redhammer, Gerold Tippelt

**Affiliations:** a University of Salzburg, Department Chemistry and Physics of Materials, Hellbrunnerstrasse 34, 5020 Salzburg, Austria

**Keywords:** crystal structure, spinel, Mössbauer spectroscopy, geoscience

## Abstract

Spinel-type Li_0.64_Fe_2.15_Ge_0.21_O_4_, lithium diiron(III) germanium tetra­oxide, has been formed as a by-product during flux growth of an Li–Fe–Ge pyroxene-type material. In the title compound, lithium is ordered on the octa­hedral *B* sites, while Ge^4+^ orders onto the tetra­hedral *A* sites, and iron distributes over both the octa­hedral and tetra­hedral sites, and is in the trivalent state as determined from Mössbauer spectroscopy. The oxygen parameter *u* is 0.2543; thus, the spinel is close to having an ideal cubic closed packing of the O atoms. The title spinel is compared with other Li- and Ge-containing spinels.

## Abstract 2

The synthetic spinel Li_0.64_Fe_2.15_Ge_0.21_O_4_ shows a partially inverse cationic arrangement with Ge^4+^ on the tetra­hedral sites and Li^+^ on the octa­hdral sites. Iron is in the trivalent state and is distributed over both type of sites.

## Chemical context   

The minerals of the spinel group are widely occurring compounds in the geosphere and are important not only in geoscience but also in many other disciplines. In recent years, in particular, Li-containing spinels like LiMn_2_O_4_ or Li_0.5_Fe_2.5_O_4_ have attracted much inter­est in battery technology as possible candidates for cathode materials in lithium ion secondary batteries (Liu *et al.*, 2014 ▸; Patil *et al.*, 2016 ▸; Thackeray *et al.*, 1983 ▸). The ideal spinel structure consists of a closed packing of anions *X*, with one-eighth of the tetra­hedral inter­stices and one-half of the octa­hedral inter­stices occupied by the cations. The vast majority of spinels crystallize in the space group *Fd* 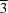 *m*. Here the cations in tetra­hedral coordination occupy special position 8*a* (point symmetry 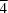
3*m*, at 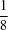, 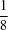, 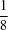), while the octa­hedrally coordinated cations reside on special position 16*d* (point symmetry 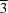 *m* at 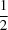, 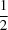, 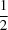). The anions are at equipoint position 32*e*, which requires one positional parameter, often denoted as the *u* parameter. For *u* = 0.25, an ideal cubic closed packing of anions is realized and the octa­hedral bond length is 1.155 times larger than the tetra­hedral one. Following Hill *et al.* (1979 ▸), variations in *u* reflect the adjustment of the structure to accommodate cations of different size in octa­hedral and tetra­hedral positions. Increasing the value of *u* above 0.25 moves the anions away along [111] from the nearest tetra­hedral cation, thereby increasing the size of the tetra­hedron at the extent of the size of the octa­hedron. The majority of the spinels can be described with the general formula *AB*_2_O_4_, with the *A* and *B* cations having the formal charges *A* = 2 and *B* = 3 (2,3 spinels) or *A* = 4 and *B* = 2 (4,2 spinels). The perfect *normal* spinel is one in which the single *A* cation occupies the tetra­hedral site and the two *B* cations reside at the two equivalent octa­hedral positions. Introducing parentheses, *i.e.* (…) and brackets, *i.e.* […], for tetra­hedral and octa­hedral coordination, respectively, one may write the normal spinels in the form (*A*)[*B*_2_]O_4_. In contrast, the complete inverse spinel has a cationic distribution of (*B*)[*AB*]O_4_ (O’Neill & Navrotsky, 1983 ▸). More detailed reviews on the spinel structure and crystal chemistry can be found, for example, in Biagioni & Pasero (2014 ▸), Harrison & Putnis (1998 ▸), Hill *et al.* (1979 ▸) and O’Neill & Navrotsky (1983 ▸).

Germanium-containing spinels are considered to belong to the normal spinels, with a full ordering of Ge^4+^ onto the tetra­hedral *A* site, while metal cations *M* order onto the octa­hedral *B* sites. This was demonstrated by, among others, Von Dreele *et al.* (1977 ▸) for GeMg_2_O_4_ and Welch *et al.* (2001 ▸) for the mineral brunogeierite (GeFe_2_O_4_). For LiMn_2_O_4_ and LiNi_0.5_Mn_1.5_O_4_, which represent excellent cathode materials, it was found that Li^+^ orders onto the tetra­hedral site (Berg *et al.*, 1998 ▸; Liu *et al.*, 2014 ▸). Also for LiCrGeO_4_, Touboul & Bourée (1993 ▸) reported an almost exclusive ordering of Li^+^ for the tetra­hedral site, while Cr^3+^ and Ge^4+^ occupy the octa­hedral sites. Different to this is the spinel Li_0.5_Fe_2.5_O_4_. This compound is an inverse spinel in which Fe^3+^ is ordered onto the tetra­hedral site, while Li^+^ and the remaining Fe^3+^ are distributed over the octa­hedral site (Hankare *et al.*, 2009 ▸; Patil *et al.*, 2016 ▸; Tomas *et al.*, 1983 ▸). This cationic distribution is thus similar to that in the inverse spinel magnetite, FeFe_2_O_4_ (Fleet, 1981 ▸).

During the synthesis of Li–Fe–Ge pyroxenes (Redhammer *et al.*, 2009 ▸, 2010 ▸), black octa­hedral-shaped single crystals were frequently obtained, which turned out to be a spinel-type compound with significant Li^+^ and small Ge^4+^ concentrations. We present here the structure refinement and ^57^Fe Mössbauer spectroscopic characterization of these crystals.

## Structural commentary   

**Figure 1:**
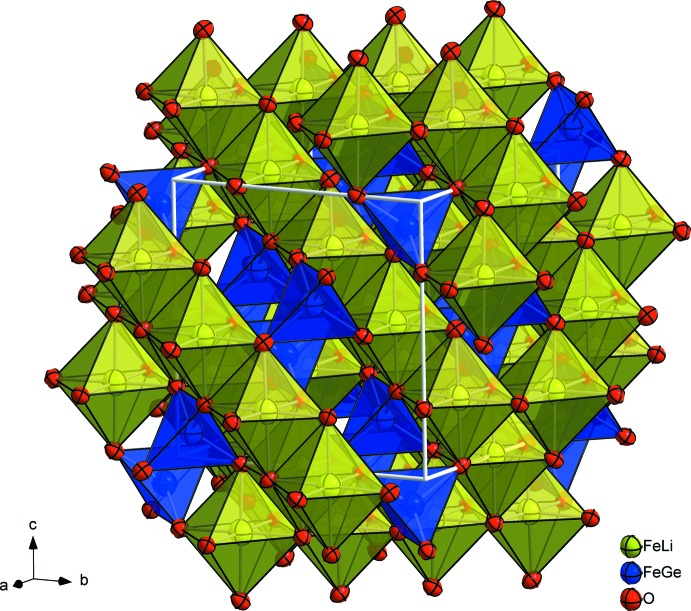
Polyhedral drawing of the spinel-type structure of the title compound. Anisotropic displacement parameters are drawn at the 95% probability level.

The structure of the title compound is shown in Fig. 1 ▸. The site-occupation refinement indicates that Li^+^ orders onto the octa­hedral *B* site, while Ge^4+^ is found on the tetra­hedral *A* site, indicating a partial inverse spinel arrangement; iron is distributed over both sites. The derived crystal chemical formula of the title compound is thus (Fe^3+^_0.79_Ge^4+^_0.21_)[Li^+^_0.64_Fe^3+^_1.36_]O_4_, with the valence state of iron determined from ^57^Fe Mössbauer spectroscopy (see below). This formula is balanced in charge and agrees very well with the chemical composition determined from electron microprobe analysis. Generally, the title compound is similar to the Li_0.5_Fe_2.5_O_4_ spinel-type materials. The shift of Li^+^ to the octa­hedral site, for example, in comparison with LiCrGeO_4_ or LiMn_2_O_4_, can be explained by the strong preference of Fe^3+^ for the tetra­hedral site. Based on the concept of crystal field stabilization energy, Miller (1959 ▸) theoretically calculated octa­hedral site preference energies which gave a stronger preference of Fe^3+^ for the tetra­hedral site as compared, for example, to Li^+^ or Mn^3+^.

The lattice parameter of the title compound [8.2903 (3) Å] is smaller in comparison with, for example, magnetite Fe_3_O_4_ [*a* = 8.3941 (7) Å; Fleet, 1981 ▸], but larger than that observed in the Li spinels LiCrGeO_4_ [*a* = 8.1976 (1) Å; Touboul & Bourée, 1993 ▸] or LiMn_2_O_4_ and LiNi_0.5_Mn_1.5_O_4_ (*a* = 8.243 and 8.1685 Å, respectively; Liu *et al.*, 2014 ▸). This is due mainly to the high amount of Fe^3+^ at the *A* sites, which has a larger ionic radius than Ge^4+^, Ni^3+^ or Mn^3+/4+^ (Shannon & Prewitt, 1969 ▸). The oxygen parameter *u* = 0.2543 is close to the ideal value for cubic closed packing, reflecting some distinct differences to the spinels which have the *A* site fully occupied by Li^+^. In the title compound, the bond length of the tetra­hedrally coordinated site *T* is 1.857 (2) Å, which is distinctly smaller than in, for example, LiMn_2_O_4_, with the tetra­hedral site being fully occupied by Li^+^. The *T*—O bond length is also smaller than in magnetite (Fleet, 1981 ▸) or Li_0.5_Fe_2.5_O_5_ (Tomas *et al.*, 1983 ▸), with values of 1.8889 (9) and 1.880 (5) Å, respectively. In GeFe_2_O_4_, the *T*—O bond length is only 1.771 (2) Å and this smaller value of *T*—O compared to, for example, magnetite is due to the substitution of Ge^4+^ onto the *A* site and can be seen as additional proof for the correctness of the derived cationic distribution.

The bond length involving the octa­hedrally coordinated site *M* is 2.0373 (11) Å, which is 1.07 times larger than the bond length involving the tetra­hedrally coordinated site. The *M*—O bond length is somewhat larger than 2.025 (3) Å in Li_0.5_Fe_2.5_O_4_ (Tomas *et al.*, 1983 ▸). This agrees well with the observed higher Li content in the title compound, with the ionic radius for Li^+^ in an octa­hedral coordination (0.740 Å) being larger than that of Fe^3+^ (0.645 Å; Shannon & Prewitt, 1969 ▸), thus increasing the *M*—O distance. Magnetite has a mixed occupation of the octa­hedral sites, with both Fe^2+^ and Fe^3+^, thus having a larger *M*—O bond length of 2.0582 (9) Å, while in GeFe_2_O_4_, all the Fe atoms are in a divalent state and an *M*—O bond length of 2.132 (2) Å is observed.

**Figure 2:**
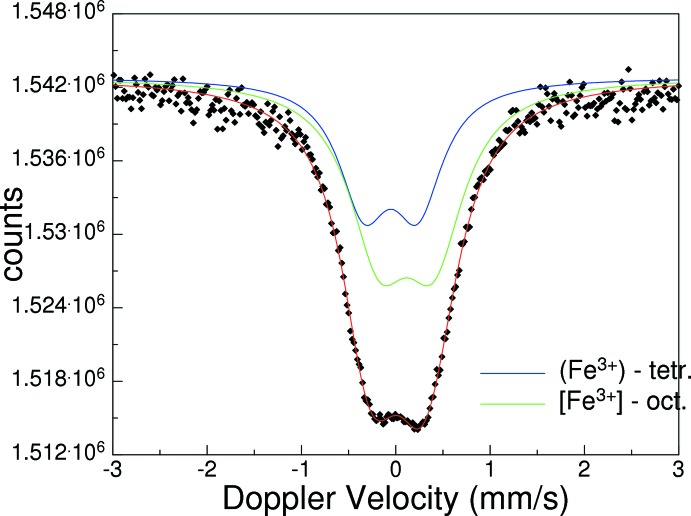
^57^Fe Mössbauer spectrum of the title compound, recorded at 740 K.

In order to qu­antify the valence state of iron in the title compound, a ^57^Fe Mössbauer spectrum was recorded at 340 K. It shows a broad, slightly asymmetric, doublet, which can be evaluated with two Lorentzian-shaped doublets (Fig. 2 ▸). The first doublet shows an isomer shift (IS) of −0.053 (17) mm s^−1^ and a quadrupole splitting (QS) of 0.57 (3) mm s^−1^, and can be assigned to the ferric iron on the tetra­hedral site. The second doublet has a larger IS of 0.115 (14) mm s^−1^ and an almost identical QS of 0.58 (2) mm s^−1^, and is assigned to ferric iron at the octa­hedral site. No indications for ferrous iron are present. The QS values suggest low polyhedral distortion, which is almost identical in both sites. The relative area ratio of tetra­hedral to octa­hedral sites is 38.6 (8) to 61.4 (9)%. Assuming a total amount of 2.15 formula units Fe^3+^, the results of Mössbauer spectroscopy give a cation distribution of (Fe^3+^_0.83_)[Fe^3+^_1.32_], which is in good agreement with that obtained from the site-occupation refinement of the X-ray data. At room temperature, the title compound is magnetically ordered, as revealed by its ^57^Fe Mössbauer spectrum.

## Synthesis and crystallization   

The spinel formed as a by-product during the synthesis of pyroxene-type LiFeGe_2_O_6_ in flux-growth experiments (Redhammer *et al.*, 2010 ▸). For the synthesis of the pyroxene, Li_2_CO_3_, Fe_2_O_3_ and GeO_2_ in the stoichiometry of the compound and Li_2_MoO_4_/LiVO_3_ as a flux (mass ratio sample to flux = 1:10) were mixed together, heated to 1473 K in a platinum crucible, covered with a lid, held at this temperature for 24 h and cooled afterwards at a rate of 1.5 K h^−1^ to 973 K. The experimental batch consisted of large pyroxene crystals and a distinct amount of black crystals with idiomorphic octa­hedral habit, up to 200 µm. Semi-qu­anti­tative EDX (energy-dispersive X-ray) analysis revealed iron and some germanium as the main elements; powder X-ray diffraction analysis revealed the crystals as a spinel-type material. An electron microprobe analysis on polished/embedded crystals (three different grains with five measurement points each) yielded a chemical composition of 84.86 (30) wt% Fe_2_O_3_, 10.52 (25) wt% GeO_2_ and 4.62 wt% Li_2_O, with the latter calculated from the difference to 100 oxide%. There is no evidence for Mo or V from the flux, nor for any other chemical elements. From the oxide percentage, a chemical formula of Li_0.63 (2)_Fe_2.18 (1)_Ge_0.20 (2)_O_4_ was calculated, which is in good agreement with that obtained from the structure refinement. Individual crystals are homogeneous in composition, with no significant systematic variation from rim-core; also, there is no systematic variation in composition from crystal to crystal.

## Refinement   

Crystal data, data collection and structure refinement details are summarized in Table 1 ▸. In a first stage of refinement, only iron was considered on the *A* and *B* sites, thereby allowing unconstrained refinement of the site-occupation factors. This gave a surplus of electron density (higher occupation than allowed by the multiplicity) at the tetra­hedral site, while a lower occupation than possible was found for the octa­hedral site. From this it was concluded that Li enters the octa­hedral site and Ge enters the tetra­hedral site. In the final refinements, it was assumed that both tetra­hedral and octa­hedral sites are fully occupied, with Fe + Ge = 1 as a restraint for the tetra­hedral site and Fe + Li = 1 for the octa­hedral site.

## Supplementary Material

Crystal structure: contains datablock(s) global, I. DOI: 10.1107/S205698901600414X/wm5279sup1.cif

Structure factors: contains datablock(s) I. DOI: 10.1107/S205698901600414X/wm5279Isup2.hkl

CCDC reference: 1463892

Additional supporting information:  crystallographic information; 3D view; checkCIF report

## Figures and Tables

**Table 1:** Experimental details

Crystal data	
Chemical formula	Li_0.64_Fe_2.15_Ge_0.21_O_4_
*M*_r_	203.5
Crystal system, space group	Cubic, *F* *d* 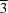 *m*
Temperature (K)	298
*a* (Å)	8.2903 (3)
*V* (Å^3^)	569.78 (6)
*Z*	8
Radiation type	Mo *K*α
μ (mm^−1^)	12.85
Crystal size (mm)	0.13 × 0.12 × 0.12

Data collection	
Diffractometer	Bruker SMART APEX CCD
Absorption correction	Multi-scan (*SADABS*; Bruker, 2012 ▸)
*T*_min_, *T*_max_	0.83, 0.94
No. of measured, independent and observed [*I* > 2σ(*I*)] reflections	3046, 118, 114
*R*_int_	0.021
(sin θ/λ)_max_ (Å^−1^)	0.940

Refinement	
*R*[*F* ^2^ > 2σ(*F* ^2^)], *wR*(*F* ^2^), *S*	0.018, 0.042, 1.37
No. of reflections	118
No. of parameters	10
No. of restraints	1
Δρ_max_, Δρ_min_ (e Å^−3^)	0.36, −0.67

Computer programs: *APEX2* and *SAINT* (Bruker, 2012 ▸), *SHELXL2014* (Sheldrick, 2015 ▸), *DIAMOND* (Brandenburg, 2006 ▸) and *WinGX* (Farrugia, 2012 ▸).

## supplementary crystallographic information

### Crystal data

**Table:**

Li_0.64_Fe_2.15_Ge_0.21_O_4_	*D*_x_ = 4.744 Mg m^−^^3^
*M**_r_* = 203.5	Mo *K*α radiation, λ = 0.71073 Å
Cubic, *F**d*3*m*	Cell parameters from 3046 reflections
Hall symbol: -F 4vw 2vw 3	θ = 7.0–41.9°
*a* = 8.2903 (3) Å	µ = 12.85 mm^−^^1^
*V* = 569.78 (6) Å^3^	*T* = 298 K
*Z* = 8	Octahedron, black
*F*(000) = 771	0.13 × 0.12 × 0.12 mm

### Data collection

**Table:**

Bruker SMART APEX CCD diffractometer	118 independent reflections
Radiation source: 3-circle diffractometer	114 reflections with *I* > 2σ(*I*)
Graphite monochromator	*R*_int_ = 0.021
ω–scan at 4 different φ positions	θ_max_ = 41.9°, θ_min_ = 7.0°
Absorption correction: multi-scan (*SADABS*; Bruker, 2012)	*h* = −15→14
*T*_min_ = 0.83, *T*_max_ = 0.94	*k* = −14→10
3046 measured reflections	*l* = −15→13

### Refinement

**Table:**

Refinement on *F*^2^	1 restraint
Least-squares matrix: full	*w* = 1/[σ^2^(*F*_o_^2^) + (0.0139*P*)^2^ + 2.542*P*] where *P* = (*F*_o_^2^ + 2*F*_c_^2^)/3
*R*[*F*^2^ > 2σ(*F*^2^)] = 0.018	(Δ/σ)_max_ < 0.001
*wR*(*F*^2^) = 0.042	Δρ_max_ = 0.36 e Å^−^^3^
*S* = 1.37	Δρ_min_ = −0.67 e Å^−^^3^
118 reflections	Extinction correction: *SHELXL2014* (Sheldrick, 2015)
10 parameters	Extinction coefficient: 0.0051 (6)

### Special details

**Table:**

Geometry. All e.s.d.'s (except the e.s.d. in the dihedral angle between two l.s. planes) are estimated using the full covariance matrix. The cell e.s.d.'s are taken into account individually in the estimation of e.s.d.'s in distances, angles and torsion angles; correlations between e.s.d.'s in cell parameters are only used when they are defined by crystal symmetry. An approximate (isotropic) treatment of cell e.s.d.'s is used for estimating e.s.d.'s involving l.s. planes.

### Fractional atomic coordinates and isotropic or equivalent isotropic displacement parameters (Å^2^)

**Table:**

	*x*	*y*	*z*	*U*_iso_*/*U*_eq_	Occ. (<1)
Fe1	0.5	0.5	0.5	0.00795 (17)	0.678 (4)
Li1	0.5	0.5	0.5	0.00795 (17)	0.322 (4)
Fe2	0.125	0.125	0.125	0.00573 (17)	0.795 (3)
Ge2	0.125	0.125	0.125	0.00573 (17)	0.205 (3)
O2	0.25434 (14)	0.25434 (14)	0.25434 (14)	0.0095 (3)	

### Atomic displacement parameters (Å^2^)

**Table:**

	*U*^11^	*U*^22^	*U*^33^	*U*^12^	*U*^13^	*U*^23^
Fe1	0.00795 (17)	0.00795 (17)	0.00795 (17)	−0.00100 (11)	−0.00100 (11)	−0.00100 (11)
Li1	0.00795 (17)	0.00795 (17)	0.00795 (17)	−0.00100 (11)	−0.00100 (11)	−0.00100 (11)
Fe2	0.00573 (17)	0.00573 (17)	0.00573 (17)	0	0	0
Ge2	0.00573 (17)	0.00573 (17)	0.00573 (17)	0	0	0
O2	0.0095 (3)	0.0095 (3)	0.0095 (3)	0.0010 (3)	0.0010 (3)	0.0010 (3)

### Geometric parameters (Å, º)

**Table:**

Fe1—O2^i^	2.0373 (11)	Fe1—Fe1^ii^	2.9311 (1)
Fe1—O2^ii^	2.0373 (11)	Fe2—O2^vii^	1.857 (2)
Fe1—O2^iii^	2.0373 (11)	Fe2—O2^viii^	1.857 (2)
Fe1—O2^iv^	2.0373 (11)	Fe2—O2^ix^	1.857 (2)
Fe1—O2^v^	2.0373 (11)	Fe2—O2	1.857 (2)
Fe1—O2^vi^	2.0373 (11)		
			
O2^i^—Fe1—O2^ii^	180	O2^ii^—Fe1—O2^vi^	87.96 (7)
O2^i^—Fe1—O2^iii^	87.96 (7)	O2^iii^—Fe1—O2^vi^	92.04 (7)
O2^ii^—Fe1—O2^iii^	92.04 (7)	O2^iv^—Fe1—O2^vi^	87.96 (7)
O2^i^—Fe1—O2^iv^	92.04 (7)	O2^v^—Fe1—O2^vi^	180.00 (7)
O2^ii^—Fe1—O2^iv^	87.96 (7)	O2^vii^—Fe2—O2^viii^	109.5
O2^iii^—Fe1—O2^iv^	180	O2^vii^—Fe2—O2^ix^	109.5
O2^i^—Fe1—O2^v^	87.96 (7)	O2^viii^—Fe2—O2^ix^	109.5
O2^ii^—Fe1—O2^v^	92.04 (7)	O2^vii^—Fe2—O2	109.4710 (10)
O2^iii^—Fe1—O2^v^	87.96 (7)	O2^viii^—Fe2—O2	109.5
O2^iv^—Fe1—O2^v^	92.04 (7)	O2^ix^—Fe2—O2	109.4710 (10)
O2^i^—Fe1—O2^vi^	92.04 (7)		

Symmetry codes: (i) *x*+1/4, *y*+1/4, −*z*+1; (ii) −*x*+3/4, −*y*+3/4, *z*; (iii) *x*+1/4, −*y*+1, *z*+1/4; (iv) −*x*+3/4, *y*, −*z*+3/4; (v) −*x*+1, *y*+1/4, *z*+1/4; (vi) *x*, −*y*+3/4, −*z*+3/4; (vii) −*x*+1/4, *y*, −*z*+1/4; (viii) *x*, −*y*+1/4, −*z*+1/4; (ix) −*x*+1/4, −*y*+1/4, *z*.
